# Purtscher’s retinopathy in one and sixth nerve palsy in the other eye following head trauma

**DOI:** 10.3205/oc000098

**Published:** 2019-03-29

**Authors:** Virna M. Shah, Gauresh Gawas

**Affiliations:** 1 Department of Neuro-Ophthalmology, Aravind Eye Hospital & Postgraduate Institute of Ophthalmology, Coimbatore, Tamilnadu, India

**Keywords:** Purtscher's retinopathy, sixth nerve palsy, simultaneous, head trauma

## Abstract

Purtscher’s retinopathy and sixth nerve palsy are often seen following head trauma. However, both are rarely seen coexisting together in the same patient. We present a rare case of both these entities occurring simultaneously in opposite eyes of the same patient following head trauma.

## Introduction

Purtscher’s retinopathy is a rare form of retinal disease seen after head trauma or chest compression injuries and was first described by Othmar Purtscher in 1910 [1]. It consists of areas of retinal whitening (Purtscher flecken) associated with superficial hemorrhages. Sixth nerve palsy is also a known entity following head trauma [2]. However, it has never been reported together. We report a case of Purtscher’s retinopathy in one eye and sixth nerve palsy in the other eye of the same patient following head trauma.

## Case description

**Figure 1:**
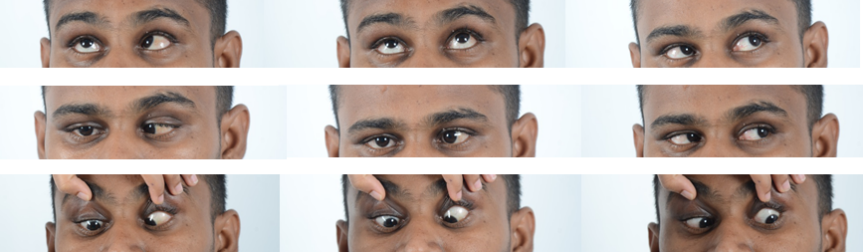
Ocular movements showing restricted abduction in the right eye

**Figure 2:**
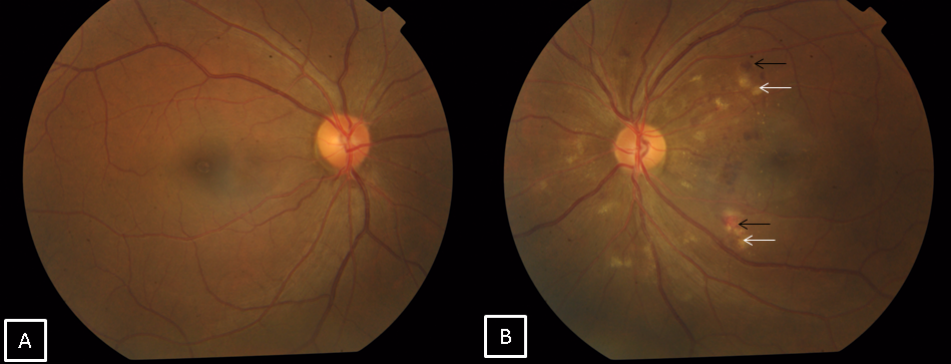
A) Normal fundus of the right eye. B) Fundus of the left eye showing multiple cotton wool spots (white arrows) with superficial retinal hemorrhages (black arrows) on the posterior pole.

**Figure 3:**
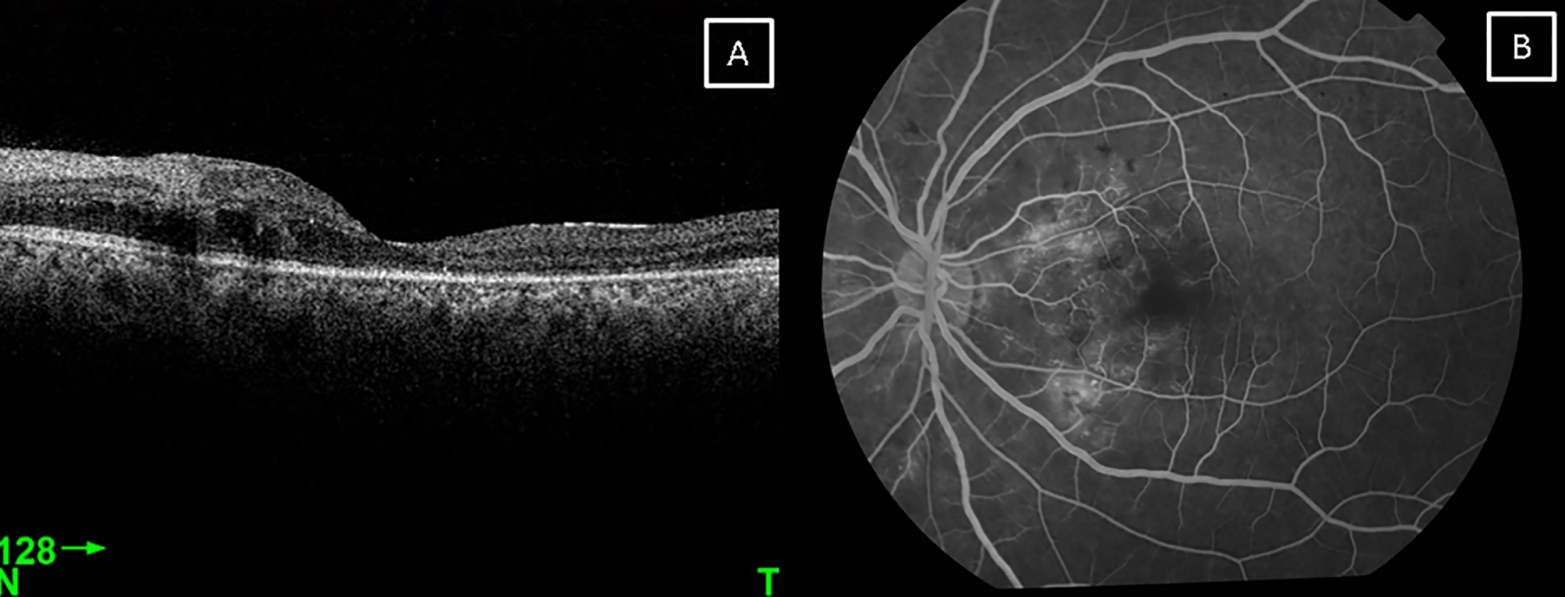
A) OCT of the left eye showing a thickening with hyperreflectivity of the inner retinal layers and retinal edema. B) Fluorescein angiography of the left eye showing blocked fluorescence corresponding to the hemorrhages, capillary non-perfusion areas with late leakage.

A 30-year-old male patient came with chief complaint of diplopia on right gaze and decreased vision in the left eye of 20 days duration following a road traffic accident. He had a fall from a two-wheeler which he was riding without a helmet. There was a history of loss of consciousness and epistaxis and he was admitted in a general hospital for four days. Computerized tomography (CT) scan of the brain showed minimal extradural bleed over the right frontal lobe with minimal subarachnoid bleed along the basal cisterns. CT scan of the orbits showed a minimally displaced fracture of the right supraorbital bone extending into the frontal bone along with a linear undisplaced fracture of the anterior and lateral walls of the right maxillary sinus. The left orbit showed an undisplaced fracture of the lateral wall along with a linear undisplaced fracture of the anterior wall of the left maxillary sinus with a hypodense collection in both maxillary sinuses. On ocular examination, his best corrected visual acuity was 20/20 in the right eye and 20/60 in the left eye. Anterior segment examination showed enophthalmos with restricted abduction in the right eye (Figure 1 (Fig. 1)) and a normal left eye. Fundus examination was normal in the right eye while the left eye showed multiple cotton wool spots surrounding the disc with superficial retinal hemorrhages (Figure 2 (Fig. 2)). Forced duction test was negative in the right eye. Color vision and contrast sensitivity were normal in the right eye but reduced in the left eye. Hess and diplopia charts showed features of right lateral rectus palsy. Optical coherence tomography (OCT) showed a thickening with hyperreflectivity in the inner retinal layers. 30-2 visual fields showed central scotoma. Fundus fluorescein angiography showed a blocked fluorescence corresponding to the hemorrhages and capillary non-perfusion areas with late leakage corresponding to the cotton wool spots (Figure 3 (Fig. 3)). The patient was managed conservatively with occlusion for one month. On follow-up examination, there was a complete resolution of the sixth nerve palsy in the right eye after two months with resolving hemorrhages and cotton wool spots in the left eye. At 20-month follow-up, visual acuity of both eyes was 20/20 with full ocular movements and minimal retinal pigment epithelium changes over the macula in the left eye.

## Discussion

Purtscher’s retinopathy was first described following blunt head trauma. It is mostly bilateral, however unilateral cases have also been reported [3]. Purtscher-like retinopathy is a similar condition seen in other systemic diseases like acute pancreatitis [4] and chronic renal failure [5] and has also been reported following uneventful cataract surgery [6]. The pathogenesis stated is a raised intracranial pressure following head injury which leads to extravasation of the lymph and in non-traumatic cases, it is hypothesized to be secondary to small emboli released due to the underlying disease [1]. Sixth nerve palsy can be due to contusion of the nerve against the petrous ridge [4]. There has been a previous case report describing Purtscher’s retinopathy with third nerve palsy in a 4-year-old child [7]. Purtscher’s retinopathy with sixth nerve palsy has never been reported. In our case, both mechanisms occurred on opposite sides and the patient presented with sixth nerve involvement in the right eye and Purtscher’s retinopathy in the left eye. Both conditions recovered well only on observation. Although it may be coincidental to have both traumatic sixth nerve palsy and Purtscher’s retinopathy in the same patient, this case shows that in a setting of blunt head trauma, a patient may present with multiple sites of ocular injury involving both eyes.

## Notes

### Competing interests

The authors declare that they have no competing interests.

### Informed consent

Informed consent was obtained from the patient for publishing his case including the photographs.
